# MR Study of Water Distribution in a Beech (*Fagus sylvatica*) Branch Using Relaxometry Methods

**DOI:** 10.3390/molecules26144305

**Published:** 2021-07-16

**Authors:** Urša Mikac, Maks Merela, Primož Oven, Ana Sepe, Igor Serša

**Affiliations:** 1 Department of Condensed Matter Physics, Jožef Stefan Institute, 1000 Ljubljana, Slovenia; urska.mikac@ijs.si (U.M.); ana.sepe@ijs.si (A.S.); 2 Department of Wood Science and Technology, Biotechnical Faculty, University of Ljubljana, 1000 Ljubljana, Slovenia; maks.merela@bf.uni-lj.si (M.M.); primoz.oven@bf.uni-lj.si (P.O.)

**Keywords:** magnetic resonance imaging (MRI), relaxation times, beech (*Fagus sylvatica*), wood, moisture content (MC)

## Abstract

Wood is a widely used material because it is environmentally sustainable, renewable and relatively inexpensive. Due to the hygroscopic nature of wood, its physical and mechanical properties as well as the susceptibility to fungal decay are strongly influenced by its moisture content, constantly changing in the course of everyday use. Therefore, the understanding of the water state (free or bound) and its distribution at different moisture contents is of great importance. In this study, changes of the water state and its distribution in a beech sample while drying from the green (fresh cut) to the absolutely dry state were monitored by 1D and 2D ^1^H NMR relaxometry as well as by spatial mapping of the relaxation times *T*_1_ and *T*_2_. The relaxometry results are consistent with the model of homogeneously emptying pores in the bioporous system with connected pores. This was also confirmed by the relaxation time mapping results which revealed the moisture transport in the course of drying from an axially oriented early- and latewood system to radial rays through which it evaporates from the branch. The results of this study confirmed that MRI is an efficient tool to study the pathways of water transport in wood in the course of drying and is capable of determining the state of water and its distribution in wood.

## 1. Introduction

Wood is a hygroscopic porous and permeable material that is widely used in everyday life. It interacts with water from humid air causing a constantly changing moisture content (MC), especially in the outdoor use where it is exposed to dynamic moisture cycles. The MC changes affect the wood properties and are responsible for shrinkage and swelling of wood, moisture-induced stresses and mechanosorptive effects, which may lead to cracking or loss of loadbearing capacity. Wood contains macromolecules that link water by hydrogen bonding [1,2]. Thus, water in wood exists as bound and free water. Free water is in the form of liquid or vapor in cell lumina and bound water is hydrogen bonded in the cell wall material. Changed in the MC in the range between the absolutely dry wood (MC = 0%) and the wood at the fiber saturation point (FSP) (approximately 30%) where all water is bound in the cell walls cause alterations in physical and mechanical properties of wood. At higher MCs, water also exists as free water with almost no effect on the physical and mechanical properties. It is established that the optimal fungal growth is achieved at MC = 35–50% on the basis of dry weight. Therefore, the knowledge about the state of water and moisture transport in wood is of utmost importance for understanding its utilization, durability and wood product quality [3].

Different methods such as traditional gravimetrical determination, methods based on the electrical properties of moist wood and titration, for instance, are used to measure the MC of wood [4]. Among other methods, nuclear magnetic resonance (NMR) and magnetic resonance imaging (MRI) have been successfully employed for studying the MC in wood [5,6,7,8,9] as well as its spatial distribution in wood samples [10,11,12,13,14,15,16,17,18,19,20]. The spin–lattice (*T*_1_) and spin–spin (*T*_2_) relaxation times of the protons in wood change with the MC. This is because the NMR relaxation times depend on the local environment of protons as they determine the mobility of molecules and thus influence the *T*_1_/*T*_2_ ratio. This ratio is higher in the environment with molecules of higher mobility [21]. The *T*_2_ of protons in solid wood is in tens of microseconds, the *T*_2_ of bound water with hindered local motion is in the range from hundreds of microseconds to several milliseconds, while the *T*_2_ of free water in the cell lumina is in the range from tens to hundreds of milliseconds [6,22,23]. In addition, the *T*_2_ values of free water depend on cell dimensions i.e., the *T*_2_ of free water is longer in cells with larger lumina [24]. Therefore, four peaks are observed in the *T*_2_ distribution of wood. The first two peaks are associated with free water and are therefore at higher *T*_2_ values. Their amplitudes decrease with a decreasing MC and they vanish at MCs below the FSP. The third peak at shorter *T*_2_ is associated with bound water. Its amplitude is constant with MCs above the FSP but it starts to decrease with MCs lower than the FSP. The fourth peak is associated with solid wood and it is at the shortest *T*_2_ values. The amplitude of this peak is constant with any MC [6,13,22,25,26]. Different relaxation time values thus enable determination of the water state in the wood. The simplest are one-dimensional (1D) *T*_1_ and *T*_2_ spectra which enable distinction between bound and free water. More complex are two-dimensional (2D) *T*_1_–*T*_2_ and *T*_2_–*T*_2_ correlation spectra with which improvement of the resolution and information on water states in the wood is significant. *T*_1_–*T*_2_ correlation spectra enable distinction between the two types of bound water in cell walls, while *T*_2_–*T*_2_ correlation spectra can identify the water exchange between cell walls and the free water in the lumina, enabling measurement of the corresponding exchange rates [27]. These methods have also been used to study the adsorption mechanisms in earlywood and latewood [28], determine the structural changes of wood after thermal modification [29] and the effect of wood aging at the molecular level [9] and to characterize the decay process of wood due to fungal decomposition [30].

Proton density-weighted MRI produces a signal that is proportional to free water in wood, but it cannot detect bound states of water and solid wood. This is because the NMR signal of bound water and solid wood decays before detection with standard imaging methods. More precise information on the state of water in wood can be obtained from *T*_1_, *T*_2_ and apparent diffusion coefficient (ADC) maps [31]. *T*_2_ maps are, in particular, important to get better contrast between free water in different wood structures [17,30].

The goal of this study is to demonstrate that NMR relaxometry is a powerful technique that allows studying the distribution and movement of water, free or bound, in different anatomical structures of wood in the course of its drying. Specifically, 1D *T*_1_ and *T*_2_ distributions, 2D *T*_1_–*T*_2_ correlation spectra and *T*_1_ and *T*_2_ maps of a beechwood sample at different MCs in the range from 90% (green state) to an absolutely dry sample were measured in this study to follow changes of the water state and distribution in the course of wood drying.

## 2. Results and Discussion

### 2.1. 1D T_1_ and T_2_ Distributions at Different MCs

**Figure 1:**
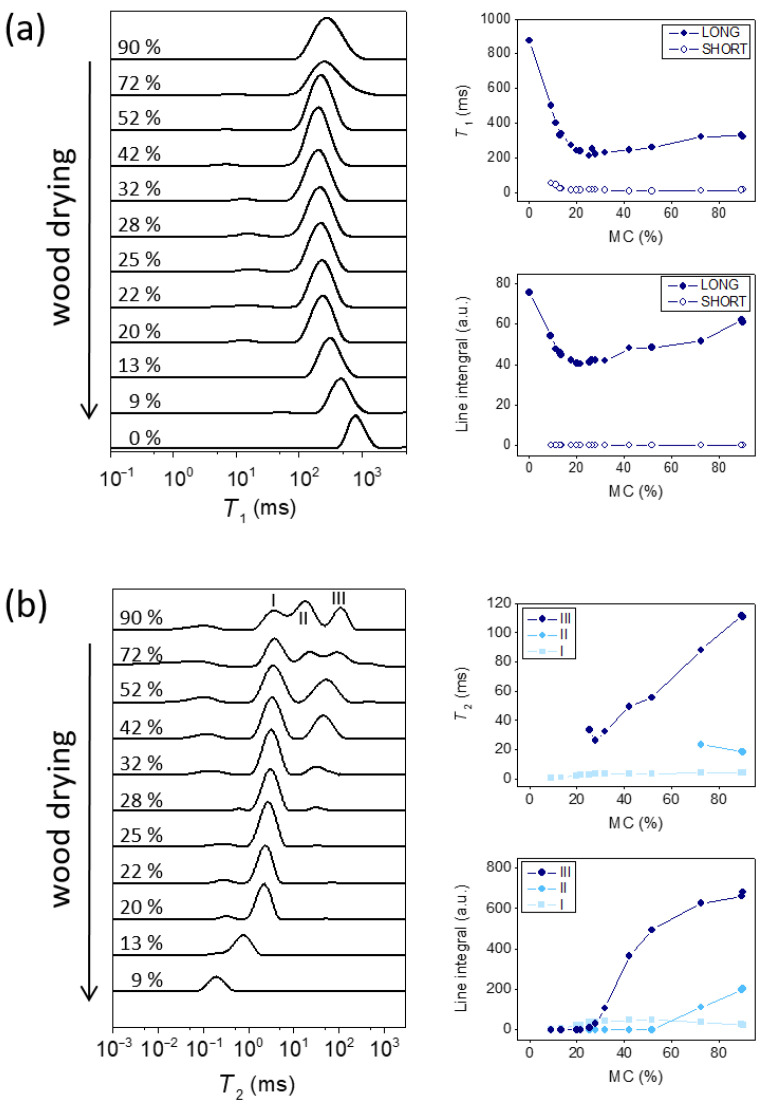
NMR relaxation time distributions together with the central relaxation time and the integrated intensities of the peaks at different MCs for the beech branchwood sample: (**a**) *T*_1_ and (**b**) *T*_2_. The labels LONG and SHORT in the graphs in panel (**a**) correspond to the *T*_1_ values of an intense peak in the range of hundreds of milliseconds (long) and to a small peak at few milliseconds (short), while the labels I, II and III in the graphs in panel (**b**) correspond to short, medium and long *T*_2_ values of three distinct peaks in *T*_2_ distributions.

A multiexponential analysis of *T*_1_ and *T*_2_ relaxation decay curves was used to determine the relaxation time distributions. Figure 1 shows the *T*_1_ and *T*_2_ distributions for different MCs. *T*_1_ distributions consisted of two peaks: an intense peak in the range of hundreds of milliseconds and a small peak at few milliseconds. With the decreasing MC (wood drying), the position of the intense peak first decreased, reached a minimum value of 210 ms at MC = 25% and then increased with the decreasing MC (Figure 1a). The values of the shorter *T*_1_ components were in the range of 10 ms. This peak was almost constant with drying until MC = 20% and then increased with a decreasing MC, up to 50 ms at MC = 9%. In the course of drying, the integrated intensities of both peaks slightly decreased until MC = 42%. Then, the integral of the longer *T*_1_ component decreased and the integral of the shorter *T*_1_ component increased in the MC range between 42% and 20%, whereas at MCs below 20%, the integral of the longer component increased and the integral of the shorter component decreased and was no longer observed at MC = 0% (Figure 1a).

The *T*_2_ distributions are, however, different (Figure 1b). A small and broad peak was observed in the *T*_2_ distribution at 0.1 ms that remained almost constant throughout the sample drying. In addition, three peaks I, II and III were observed at higher MCs. With the decreasing MC (wood drying), peak I remained at the same position until MC = 20% and shifted to lower values at lower MCs. The *T*_2_ of peak II slightly increased when MC decreased from 90% to 72% and then overlapped with peak III or I at lower MCs. Peak III shifted to lower values with the decreasing MC. The integrated intensity of peak I increased with the decreasing MC until 52%, remained constant until MC = 25% and decreased with MC further decreasing, while the integral of peak III decreased with the decreasing MC and was no longer observed at MCs lower than 25%. As in the previous studies [25,26,29,30,32,33,34,35], the peaks I, II and III were assigned to bound water, free water in cells with smaller lumina and free water in cells with larger lumina, respectively.

### 2.2. Two-Dimensional T_1_–T_2_ Correlation Spectra at Different MCs

**Figure 2:**
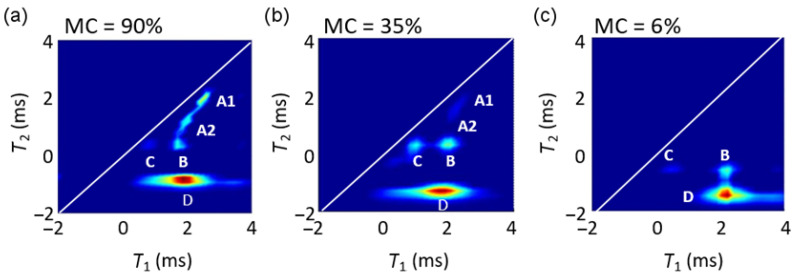
Two-dimensional *T*_1_–*T*_2_ correlation spectra of the beech branchwood sample at: (**a**) MC = 90%, (**b**) MC = 35% and (**c**) MC = 6%. The five peaks are attributed to free water in cell lumina (A1 and A2), protons of bound water (B and C) and solid wood protons (D) as discussed in the text.

To further evaluate the *T*_1_ and *T*_2_ results, 2D *T*_1_–*T*_2_ correlation spectra were measured for three different MCs (Figure 2). At MC = 90%, five peaks (labeled A1, A2, B, C and D, see Figure 2) were observed, with two different *T*_1_ and four different *T*_2_ values. The peaks A1, A2 and C were just below the diagonal *T*_1_ = *T*_2_, while the peaks B and C had similar *T*_2_ but different *T*_1_. The intensities and positions of the peaks kept changing with MC. At MC = 35%, intensities of the peaks A1 and A2 decreased significantly and could not be distinguished, and the intensity of peak C increases compared to its intensity at MC = 90%. At MC = 6%, the peaks A1 and A2 were no longer observed, and peak C had a very low intensity. The *T*_1_ values of all the peaks decreased when the MC decreased from 90% to 35% and increased again when the MC decreased to 6%. Peaks B and C had similar *T*_2_ values at MC = 90% and 35%; however, their *T*_2_ values decreased at MC = 6%. The peaks in the *T*_1_–*T*_2_ correlation spectra could be identified on the basis of previous analyses [27]. The peaks with longer *T*_1_ and the longest *T*_2_ (A1 and A2) arose from water with the highest molecular mobility, i.e., free water in lumina with different diameters. Peak B with shorter *T*_2_ corresponded to bound water, peak D with the shortest *T*_2_—to solid-like protons. Peak C with shorter *T*_1_ and the same *T*_2_ as peak B was assigned to the water absorbed in wood polymers.

### 2.3. MR Imaging: Proton Density Images and T_1_ and T_2_ Maps

The spatial distributions of relaxation times in the wood sample were measured by *T*_1_ and *T*_2_ mapping. For the proton density imaging, the first image with the shortest echo time of a sequence of echo images for *T*_2_ map determination was used. It should be noted that the shortest echo time was still too long to allow detection of a signal from protons in solid wood as their *T*_2_ values are in the range of tens of microseconds. The imaging method which was used allows detection of signals with *T*_2_ values over a millisecond. For the same reason, the signal of bound water with *T*_2_ of hundreds of microseconds produces a low signal that is, therefore, not completely detected. Thus, the signal of proton density images consists mainly of free water. Relaxation time maps were calculated by the complete set of echo images using the best fit to the monoexponential decaying function. The resolution of the images is lower than the size of a wood cell and therefore each pixel of the image consists of several cells with the cell lumina and cell walls. This implies that the multi-component decaying exponential function would yield a more accurate fit to the data and determine the relaxation times of all the states of water and solid protons in each pixel. However, due to the insufficient signal-to–noise ratio (SNR), the monoexponential fit was used. In addition, *T*_2_ values measured using the spin-echo imaging pulse sequence at various echo times are underestimated due to diffusional loss of the signal during read gradients [31,36]. Therefore, the *T*_2_ values cannot be directly compared to the spectroscopically determined *T*_2_ values, especially for protons with longer *T*_2_ values. Nevertheless, the *T*_1_ and *T*_2_ maps can still give valuable information on the water in different wood structures.

**Figure 3:**
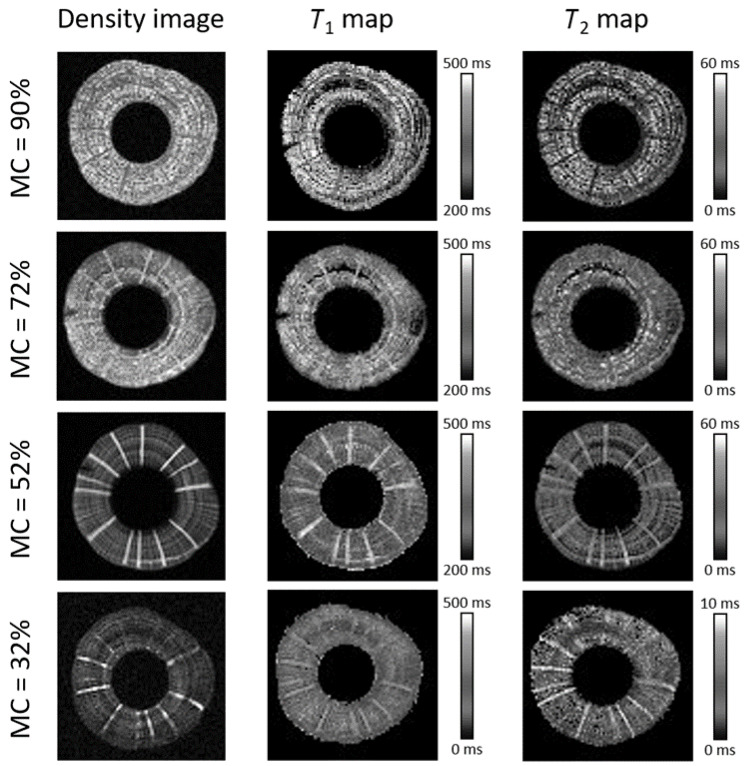
Density images, *T*_1_ and *T*_2_ maps of beech branchwood at different MCs. Note that the scales for the *T*_1_ and *T*_2_ maps are different for MC = 32% than for the higher MCs.

Proton density images, *T*_1_ and *T*_2_ maps are shown in Figure 3. The brightness of these images is proportional to proton density, *T*_1_ and *T*_2_ relaxation times, respectively. The proton density image at MC = 90% shows different anatomical structures: annual rings with earlywood and latewood and rays. The annual rings and rays are also clearly shown on the *T*_1_ and *T*_2_ maps. It can be seen from the maps that both relaxation times were longer in the earlywood compared to the latewood and the shortest in the rays (Table 1). As the MC decreased, the contrast between different wood tissues increased. Signal intensity in the rays increased due to an increased amount of free water with longer *T*_2_ relaxation time. In contrast, the signal of the annual rings decreased due to a decrease of free water amount as well as *T*_2_ reduction in partially filled lumina. At MC = 32%, the rays were either filled with free water or already empty, which can be seen in the corresponding MR image and maps as indicated by high or no signal intensity.

In some MR maps, a dark region with shorter *T*_1_ and *T*_2_ values or even with non-defined relaxation times values is observed due to too low SNR. It is interesting to note that the relaxation times of the rays in this region remained the same as for the rays elsewhere in the sample. This region is not observed in images at all the MCs because the sample was removed from the magnet after measurement at each MC, and the slices of the images at different MCs might be slightly different.

### 2.4. Discussion

Wood contains two main proton compartments: solid wood material (cellulose, hemicellulose and lignin) and water that can be observed in cell cavities as lumen water (free water) or bound in cell walls (bound water). It should be noted that the relaxation times of lumen water depend on the cell size [1]. Since wood generally contains a continuous distribution of cell sizes, the analysis of relaxation time distributions using inverse Laplace transformation (LT) is more appropriate than a multiexponential analysis using a model function equal to the sum of a predefined number of exponentially decaying functions. In the study, 1D inverse Laplace transformation was applied to the experimental data obtained by the inversion recovery (IR) and Carr–Purcell–Meiboom–Gill (CPMG) pulse sequences to calculate 1D distributions (spectra) of the *T*_1_ and *T*_2_ relaxations times, respectively. The drawback of the 1D LT relaxation time distribution analysis is that it cannot always resolve all different proton compartments in wood, particularly in cases when different proton compartments have similar *T*_1_ or *T*_2_ values and the spectral peaks overlap. However, if these protons have similar *T*_2_ but different *T*_1_ values or vice versa, then it is possible to resolve these different proton compartments by 2D *T*_1_–*T*_2_ correlation spectroscopy. This was performed using 2D LT of the data acquired by the IR–CPMG sequence. Two-dimensional *T*_1_–*T*_2_ correlation spectra were measured at three different MCs in order to differentiate the overlapping peaks in the 1D relaxation time spectra. To obtain differences in relaxation times for different wood structures, 2D *T*_1_ and *T*_2_ maps were measured as well.

The *T*_1_ distributions had two peaks (Figure 1a). The two peaks in the *T*_1_ distributions were attributed to different *T*_1_ values of earlywood and latewood in red cedar and hemlock [37] or the fast exchange between free and bound water [25,27]. Results of the *T*_1_ maps (Figure 3, Table 1) yielded values in the earlywood, latewood and ray regions in the range of the longer *T*_1_ component, i.e., 100–700 ms. These results are therefore more consistent with the fast exchange scenario.

Differences in the *T*_2_ distributions (Figure 1b) in the course of sample drying show that *T*_2_ and integrated intensity of peak III decreased with the decreasing MC and the peak vanished at MC = 25%. This value is close to that of the FSP where all free water evaporates and only bound water remains. Peak III can therefore be assigned to free water in cell lumina. Peak II could not be distinguished from peaks I and III at MCs below 72%. It is interesting to note that the *T*_2_ value of peak II increased when MC decreased from 90% to 72%. In the previous studies, these two peaks were associated with free water in cell lumina of different sizes [25,29,32,33,34,35] as the *T*_2_ value is directly proportional to the pore size [24]. Peak III was assigned to free water in earlywood vessels, peak II—to free water in smaller latewood vessels and ray cells. Another study suggested that peak III corresponds to free water in tracheid (fiber) cells, peak II—to free water in ray cell lumina, pits and tracheid lumen ends [32]. The *T*_2_ value of peak I was constant down to MC = 20% (just below the FSP) and then decreased with the decreasing MC. The dependence of the integrated line intensity of peak I on MC is interesting. The integrated intensity first increased, then it was constant and finally decreased again below MC = 20%. This can be explained by the model of a bioporous system with connected pores [38]. The *T*_2_ value and the integrated intensity of peak III decreased in the course of drying indicating the homogeneous decrease of water in large pores. The increase in the integrated intensity of peak I shows that the larger and smaller pores were connected, and emptying of the large pores left some liquid films along the walls. The water of the liquid film has a much shorter *T*_2_ that could overlap with the *T*_2_ values of the smaller pores or even with the *T*_2_ values of bound water. This result was also supported by the *T*_1_–*T*_2_ correlation spectra (Figure 2) where two peaks with different *T*_2_ values and an identical *T*_1_ value were observed, i.e., peaks B and C. The intensity of peak C, i.e., the bound water with higher mobility (higher *T*_1_/*T*_2_) increased as the MC decreased from 90% to 35% than the bound water assigned to peak B (lower *T*_1_/*T*_2_), At MC = 35%, almost no free water was in the cell lumina (low intensity of the peaks A1 and A2). This result can be explained by an increasing proportion of liquid film on cell walls with decreasing MCs (wood drying). The signal of the liquid film can be assigned to the peak of bound water with higher mobility (peak C). With further drying of the sample below the FSP, the intensity of peak C decreased while the intensity of peak B was almost the same, i.e., highly mobile bound water evaporates first, causing the decrease of peak II at MCs below 20%.

In addition to three peaks (I, II and III) in the *T*_2_ distribution, there was also a peak at much shorter *T*_2_ values of around 100 µs corresponding to peak D in the *T*_1_–*T*_2_ correlation spectra. This peak remained constant throughout drying of the sample and was assigned to solid wood. However, the *T*_2_ values of the solid wood are in the range of several tens of microseconds. This is too short for signal detection with the CPMG sequence at the parameters used in this study. Thus, most probably only the part of the spectrum with the longest *T*_2_ values of solid wood was successfully measured while the actual *T*_2_ of this peak was below our detection limit.

The spatial distribution of the *T*_2_ value at various MCs is shown in *T*_2_ maps (Figure 3 and Table 1). Shorter *T*_2_ value for latewood than for earlywood at all MCs was observed, which is in agreement with a previous study [17] and is the consequence of larger lumina of earlywood cells compared to latewood cells. The *T*_2_ value of the rays first increased with the MC decrease down to 52%. At this MC, the *T*_2_ value of the ray tissue was even higher than the *T*_2_ value of earlywood. As the MC decreased to 32%, the *T*_2_ value of rays decreased as well but was still higher than the *T*_2_ value of larger earlywood vessels at this MC. The multiseriate rays were larger than the earlywood vessels. Therefore, an additional reason for the longer *T*_2_ value was a higher amount of water in ray cells; namely, the *T*_2_ value increased with the water concentration in pores [38]. These results indicate that in the course of drying of a wood sample, water is diffused from the annual rings to the rays before evaporating from the sample. The spatial distribution of the *T*_2_ value at different MCs can also explain vanishing of peak II below MC = 72%, i.e., high above the FSP. The *T*_2_ value of the ray cells at high MC contributed to peak II. As the ray cells were filled with more water at lower MCs, the *T*_2_ of free water in the cells increased and began to overlap with the *T*_2_ value of peak III. Free water in latewood cells also contributed to peak II at high MCs. However, as the amount of water decreased in the course of drying, the *T*_2_ value of the latewood decreased to several milliseconds such that the *T*_2_ value of free water in partially empty latewood cells could overlap with peak I.

This study was performed on small samples due to the sample size limitations of the MRI scanner that was used in the study. The scanner was optimized for spatial resolution (for MR microscopy) and therefore had very sensitive but small RF probes. The largest RF probe had a diameter of only 27 mm and this was also the largest sample size that could be scanned. However, the identical methodology used in this study can be used on a much larger scale, e.g., with clinical scanners, where the samples can be up to ten times larger than in this study.

## 3. Materials and Methods 

### 3.1. Plant Material

Five 15-mm-long samples of a young forest beech tree (*Fagus sylvatica L.*) were cut from fresh branches with a diameter of approximately 8 mm and the annual growth ring width of 0.2 mm. Pith and bark were removed from the samples to avoid large variations of MCs in the samples. The samples were then dried in a desiccator until the MC of the samples decreased from the initial 88% (in the green state) to below 20%. This was needed in order to reach the state of wood below the fiber saturation point (FSP) with only bound water. To moisten the samples to different well-defined MCs, they were equilibrated in a desiccator over different salt solutions ensuring different relative air humidities (RH): MgCl_2_ (RH = 33%), K_2_CO_3_ (RH = 44%), NaNO_2_ (RH = 65%), NaCl (RH = 75%), and ZnSO_4_ (RH = 85%). After all the MR experiments were finished, the samples were completely dried in the oven at 103 °C for several hours until their masses were equilibrated. The MCs were determined gravimetrically using the Equation (1).

(1) $$\mathrm{MC}=\frac{m-m_{0}}{m_{0}}\times 100\%$$

where *m* is the mass of a moist sample and *m*_0_ is the mass of an absolutely dry sample. Wood density in the absolutely dry state was 580 kg/m^3^.

### 3.2. NMR and MRI Experiments

The NMR and MRI experiments were performed on a system consisting of a superconducting 2.35-T (^1^H NMR frequency of 100 MHz) horizontal bore magnet (Oxford Instruments, Abingdon, UK) equipped with gradients and RF coils for MR microimaging (Bruker, Ettlingen, Germany) using a Tecmag Apollo (Tecmag, Houston, TX, USA) NMR/MRI spectrometer. For the MR experiments, the wood sample was taken out of the desiccator at appropriate time intervals, weighted and inserted into a glass tube that was sealed with a Teflon cap to prevent sample drying during the scanning. The sample was reoriented in the magnet in such a way that it allowed the imaging of an axial slice (parallel to the radial–tangential plane) in 2D MRI experiments. Each sample was weighted before and after the MR measurements. The maximal change of weight during MR experiments was less than 2% and observed only for the samples with high MC, while the mass differences were negligible for the samples with MCs less than 30%.

The spin–spin relaxation times *T*_2_ were measured using the Carr–Purcell–Meiboom–Gill (CPMG) sequence 90°–τ–[180°–τ–AQ–τ]^N^ with the echo time τ of 150 µs and loop repetitions *N* of 3000 in order to enable measurement of a wide range of *T*_2_ values for the sample with different MCs. To measure the spin–lattice relaxation time *T*_1_, the inversion recovery (IR) pulse sequence 180°–τ_1_–90°–AQ was used, with the logarithmically increasing IR delay τ_1_ (from 20 μs to 10 s; 36 different τ_1_ values). To further validate the relaxation results, 2D *T*_1_–*T*_2_ relaxation correlations were measured at three different MCs, 90%, 35% and 6%, using the IR-CPMG sequence, where the IR part was followed by the CPMG loop [39]. The IR delays were the same as for 1D *T*_1_ measurements. The echo delays in the CPMG loop were equal to 350 μs, 50 μs and 25 μs, with the number of loops of 2048, 1024 and 512 for the samples with the MC of 90%, 35% and 6%, respectively.

The experimental data of *T*_1_, *T*_2_ and *T*_1_–*T*_2_ measurements were processed via a multiexponential analysis using the Prospa software that was provided by Prof. P. Callaghan [36,39]. The analysis based on multidimensional inverse Laplace transformation allows the resolution and quantification of various components in the relaxation distribution to some extent.

Two-dimensional *T*_1_ and *T*_2_ relaxation time maps were measured using a modified spin-echo imaging pulse sequence. Specifically, the inversion recovery spin-echo (IR-SE) imaging sequence was used for *T*_1_ mapping, i.e., a hard 180° pulse followed by the time interval τ_1_ added before the standard 2D spin-echo imaging sequence. *T*_1_ maps were determined from the IR-SE images measured with the time interval τ_1_ ranging from 40 μs to 10 s (nine different τ_1_ values); the echo time was equal to TE = 3.6 ms and the repetition time was TR = 10 s. *T*_2_ maps were determined from the standard 2D spin-echo images measured with the echo time (TE) varying between 3.6 ms (the shortest possible TE) and 300 ms (nine different values). The other imaging parameters for 2D images were as follows: field of view (FOV) = 13 mm, matrix size of 128 × 128 and slice thickness = 1 mm with the in-plane resolution of 0.1 mm. Proton density-weighted images were selected as the images with the shortest echo time (TE = 3.6 ms) of the sequence used for *T*_2_ map calculation.

## 4. Conclusions

The present study demonstrates that a combination of 1D *T*_1_ and *T*_2_ spectra, 2D *T*_1_–*T*_2_ correlation spectra and their spatial distributions given by the *T*_1_ and *T*_2_ maps provides valuable information about changes in wood in the course of drying. The obtained results enabled precise analysis of moisture redistribution in the course of drying between different anatomic regions of wood. It also enabled determination of the ratio between the amounts of bound and free water as well as the amount of water in wood cells of different lumina. The advantage of the proposed method is also that it is non-destructive, non-invasive and non-contact and therefore enables MC analysis of the same sample during different stages of its drying.

## Figures and Tables

**Table 1:** *T*_1_ and *T*_2_ values of earlywood, latewood and ray regions obtained from the *T*_1_ and *T*_2_ maps at different MCs.

MC		*T*_1_ (ms)	*T*_2_ (ms)
90%	Earlywood	350 ± 30	35 ± 5
	Latewood	290 ± 20	17 ± 2
	Ray	290 ± 20	12 ± 2
72%	Earlywood	330 ± 30	30 ± 5
	Latewood	300 ± 20	17 ± 2
	Ray	400 ± 20	27 ± 2
52%	Earlywood	320 ± 30	24 ± 3
	Latewood	260 ± 20	13 ± 2
	Ray	400 ± 20	35 ± 2
32%	Earlywood	230 ± 30	5 ± 1
	Latewood	180 ± 20	3 ± 1
	Ray	280 ± 20	8 ± 1

## Data Availability

The data presented in this study are available on request from the corresponding author.

## Sample Availability

Not available.
